# Influence of Lactobionic Acid on Hydration and Elasticity Parameters in Women Aged 30–40 and 50–60 Years in Comparison to Mandelic Acid

**DOI:** 10.3390/jcm14051619

**Published:** 2025-02-27

**Authors:** Marlena Warowna, Agnieszka Strzelecka, Beata Kręcisz

**Affiliations:** 1 Beauty Science Department, Faculty of Health Sciences, Vincent Pol University in Lublin, ul. Choiny 2, 20-816 Lublin, Poland; 2 Collegium Medicum, Jan Kochanowski University of Kielce, Al. IX Wieków Kielc 19A, 25-317 Kielce, Poland

**Keywords:** lactobionic acid, mandelic acid, skin, skin elasticity, hydration skin

## Abstract

**Background/Objectives**: Mandelic acid as a chemical peel has been used in cosmetology for years and is being gradually exchanged for lactobionic acid. However, in the cosmetology literature, there is no information on the effectiveness of the aforementioned peels in terms of hydration and elasticity for women’s facial skin depending on their age. The effects of lactobionic and mandelic acid on the skin of women aged 30–40 and 50–60 years are assessed in terms of their moisturizing and elasticizing effects. **Methods**: The participants of this study were 180 women aged 30–40 and 50–60 years. The selection of the group was random. Contraindications to the examination were excluded in all participants. The level of hydration was assessed using Tewameter and the level of elasticity using a cutometric probe (Courage-Khazaka Multi Test Skin Center MC 1000, Kőln, Germany). The participants also used a moisturizer. Moreover, a series of five exfoliating treatments using 40% mandelic acid or 40% lactobionic acid were performed in 120 of the participants. After 14 days since the last treatment, the assessment of their facial skin’s hydration and elasticity was performed yet again. **Results**: In the case of women aged 30–40 years, the level of hydration and elasticity increased after using lactobionic and mandelic acid, as well as a moisturizer. In the group of older women aged 50–60 years, the level of elasticity increased after using both acids and moisturizer, but the hydration parameter increased only after the usage of lactobionic acid and moisturizer. **Conclusions**: In terms of age, lactobionic acid will be more suitable for people aged 50–60, while for women aged 30–40, lactobionic or mandelic acid can be used.

## 1. Introduction

In recent years, more interest in cosmetic treatments has been observed. While planning procedures, special attention should be paid to the patient’s age, and a deep analysis of facial skin biophysical parameters should be performed. Only then can a specialist choose the appropriate treatment for a patient’s skin. To date, cosmetologists have carried out visual and palpatory assessments of the skin using a magnifier lamp or Wood’s lamp. However, these are subjective methods and rely heavily on the experience of the person performing the analysis. Nowadays, in many reputable beauty salons, diagnoses are carried out using specialized probes that objectively assess the level of biophysical parameters of the facial skin [1].

Chemical peels have been used in cosmetology for years. The downside of such cosmetics has been their irritating and erythematous effects. Over time, minimally invasive measures have been sought to prevent disruptions to patients’ lives and would be well tolerated by the skin. Mandelic acid is a preparation well-known on the cosmetic market but has gradually been exchanged for lactobionic acid, due to its advantage of not causing skin irritation after treatment [2,3,4].

Lactobionic acid (C_12_H_22_O_12_) consists of one gluconolactone molecule and a D-galactose molecule. It contains eight hydroxyl groups that strongly bind to water and belongs to the group of polyhydroxy acids (PHAs). Lactobionic acid is naturally obtained by the oxidation of lactose [5,6].

Lactobionic acid has all the features and properties characteristic of alpha-hydroxy acids (e.g., glycolic acid), but unlike them, it does not cause side effects in the form of intense peeling, burning, or redness of the skin. It should be emphasized that lactobionic acid can be used all year round in the form of cosmetics or exfoliation treatments because it does not cause phototoxic or photosensitizing effects. Preparations with lactobionic acid have strong oxidative properties, thanks to which they gently penetrate the skin without causing irritation [5,7].

Lactobionic acid is a strong inhibitor of matrix metalloproteinases, which contribute to the degradation of extracellular matrix components, resulting in loss of skin elasticity, the appearance of wrinkles, and telangiectasia [8].

Lactobionic acid is also used not only in cosmetology but also in dermatology for the care of vascular skin as well as in the treatment of rosacea, psoriasis, and seborrheic dermatitis. Preparations with lactobionic acid can be applied alone or as a complement to pharmacological treatments. During treatment with retinoids or steroids, it is recommended to use lactobionic acid, as it reduces the side effects of the above-mentioned drugs. It can also be used after invasive procedures that involve the use of high-percentage chemical peels, laser therapy, or needle mesotherapy [8,9,10].

Mandelic acid (C_8_H_8_O_3_) belongs to the group of optically active α-hydroxy acids containing an aromatic group. It occurs in the form of D- and L-enantiomers of mandelic acid. It is a white substance in the form of crystalline flakes that darkens when exposed to light. Natural sources of mandelic acid include bitter almonds (*Prunus amygdalus var. amara*), cherry seeds (*Prunus cerasus*), and apricot seeds (*Prunus armeniaca*) [10].

Exfoliating care treatments using mandelic acid have been popular in beauty salons for many years. When applied at a lower concentration, mandelic acid weakens the mutual adhesion of corneocytes, as a result of which the keratinized epidermis is removed. A higher concentration of the acid and its low pH cause epidermolysis due to the splitting of the desmosomal connections of the cells in the basal layer of the epidermis [11,12,13].

During the exfoliation of individual layers of the epidermis, the proliferation of keratinocytes in the basal layer is stimulated, and in the dermis, the processes of collagen and intracellular matrix production are intensified. This acid, in addition to its exfoliating effect due to the loosening of ionic bonds, also has a moisturizing effect thanks to its stimulation of the synthesis of ceramides in the epidermis [11,12,13].

Mandelic acid is a delicate peel that does not cause skin irritation. It can be used to care for all skin types, regardless of season, because it does not increase sensitivity to UV rays. Almond peeling treatments have a metabolic effect and lead to cell renewal, which in turn affects the synthesis of collagen and elastin fibers in the dermis [14,15,16].

A review of the literature indicates that there are few objectified studies confirming the effect of chemical peels on the biophysical parameters of facial skin. Therefore, a study was undertaken to evaluate the efficacy of chemical peels based on measurements of the biophysical parameters of the facial skin of age-matched women who received a series of exfoliating treatments with mandelic and lactobionic acid.

## 2. Purpose of This Work

Despite numerous cosmetic and dermatological indications for the use of lactobionic and mandelic acid, there are no objective studies confirming the beneficial effect of these treatments on the biophysical parameters of facial skin, depending on the age of the clients. Moreover, there are no studies comparing the results of biophysical measurements of facial skin after a series of treatments using lactobionic and mandelic acids.

Therefore, the aim of the study is to assess the effect of lactobionic and mandelic acid on the facial skin of women aged 30–40 and 50–60 in terms of moisturizing and elasticizing effects.

## 3. Materials and Methods

### 3.1. Test Procedure

Any woman who met the inclusion criteria and did not meet the exclusion criteria could join the study.

The inclusion criteria included written, informed consent to participate in the study and the age range of 30–40 or 50–60 years. The exclusion criterion from the study was related to the lack of consent to participate in the study, non-compliance with adopted age ranges and contraindications, i.e., allergy to components of the preparation, excessive exposure to the sun before the planned procedure, sun irritation, oral retinoid therapy (within 6 months), active bacterial, viral, or fungal infection, facial surgery (within 6 months), chemotherapy, severe inflammatory process in the body, skin cancer, e.g., malignant melanoma, pregnancy and lactation, atopic dermatitis, fresh scars, disruption of the epidermis, atypical moles, and severe forms of acne vulgaris, e.g., keloid acne, pyogenic acne.

**Figure 1:**
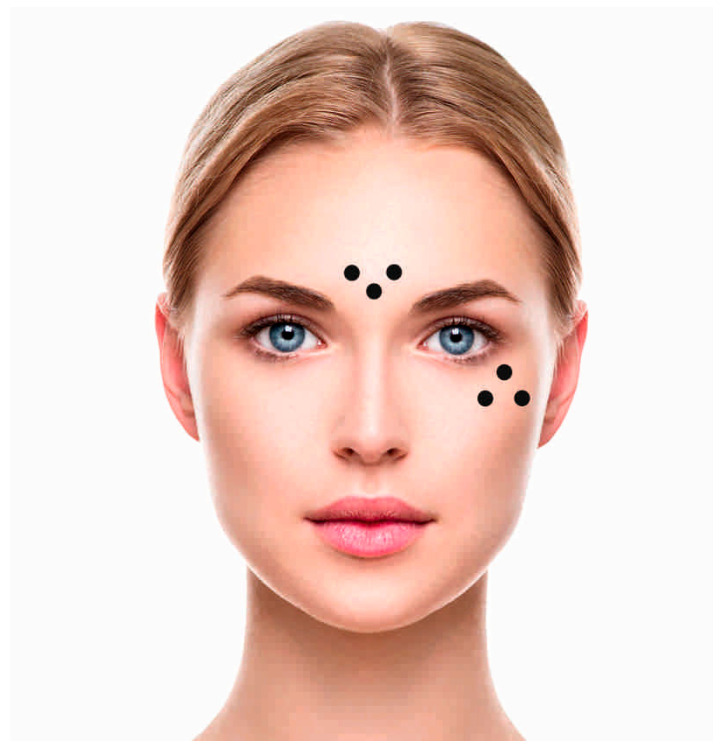
Points of application of heads for reading biophysical parameters in two areas of facial skin (medial and lateral part).

In the initial stage of the study, the participants were randomly divided into a study group of 120 people and a control group of 60 people. A short survey was conducted with women, which excluded any contraindications to exfoliation treatments, and they were asked for written consent to participate in the study. Then, using the Courage + Khazaka Multi Skin Test Centre MC 1000 (Kőln, Germany) diagnostic apparatus equipped with probes for hydration (coneometer^®^) and elasticity (cutometer^®^), biophysical parameters were measured on the right cheek (at 3 adjacent points) and in the central part of the forehead (also at 3 points thereof) (Figure 1). The apparatus then calculated the arithmetic mean, which constituted the final result of the examined facial skin parameter. The women participating in the study were asked to come to the study without make-up. 

Biophysical parameters were measured after 15 min of acclimatization in a sitting position, in a room with a temperature of approximately 21 °C and room humidity ranging between 40–60%.

In the next stage of the study, a series of 5 exfoliating treatments using chemical peels were performed on 120 participants (study group)—60 women with 40% mandelic acid (pH 1.5 and pKa 3.41), including 30 aged 30–40 years and 30 aged 50–60 years, and 60 women with 40% lactobionic acid (pH 1.9 and pKa 3.8), in the respective age ranges at 7-day intervals. The choice of a particular peel for every woman taking part in the study was random. In addition, volunteers were asked to use the indicated moisturizing cream in the morning and evening (which included shea butter, d-panthenol, and silicones) and, when going out into the sun, to spray with sunscreen. Women also received guidelines on how to care for their skin during the study period (e.g., do not use other preparations than those indicated) and were stressed not to use the sun, swimming pools, or saunas. At 14 days after the last peel, the biophysical parameters of the skin were reassessed. The overall duration of the study for individuals was approximately 5 weeks. In the control group, a reassessment of the biophysical parameters of the facial skin was performed after 7 weeks of application of the moisturizer.

The project was approved by the Faculty Bioethics Committee of Jan Kochanowski University in Kielce (36/2018 dated 8 June 2018).

### 3.2. Statistical Analysis

The statistical methods used in the study depended on the type of variables analyzed. Among others, the parametric Student’s *t*-test for dependent groups and the non-parametric (in the case of the non-normality of the distribution of the studied variables), the Shapiro–Wilk test, for repeated measures the non-parametric ANOVA test was applied (also the normality of distributions was verified using the Shapiro–Wilk test), while for the determination of observed values, a Configuration Frequency Analysis (CFA) was performed, which was based on the expected values of a given combination of variables from the values of the standardized normal distribution.

## 4. Results

The analysis of biophysical parameters of facial skin conducted before the series of treatments/using moisturizer showed that younger as well as older women had slightly dehydrated skin, and their average hydration was, respectively, 58.34 CK and 62.70 CK, and the level of their elasticity in both groups was within the proper range.

In the group of women aged 30–40 years, after the series of treatments, the skin hydration improved – after the use of mandelic acid by 6.65 CK, after the use of lactobionic acid by 14.11 CK, and after the use of moisturizer by 9.52 CK (Table 1).

In the group of women aged 30–40 years, the elasticity improved after using both peels and moisturizer. After using mandelic acid, elasticity improved by 8.62 CK, lactobionic acid by 5.84, and moisturizer by 3.46 CK (Table 2).

In the group of women aged 50–60 years after the series of treatments, the hydration improved only after using lactobionic acid (by 11.32 CK) and moisturizer (by 3.34 CK) (Table 3).

The used cosmetics improved the elasticity—after using mandelic acid the elasticity improved by 6.54 CK, lactobionic acid by 7.39 CK, and moisturizer by 3.98 CK (Table 4).

After completing a series of treatments in the study group and applying the cream in the control group, an interview was conducted with volunteers regarding possible side effects of the procedures used. None of the women experienced irritation or other undesirable symptoms, such as redness, burning, itching, or rash. It should be emphasized that in our own studies, preparations were safe and well tolerated.

## 5. Discussion

### 5.1. Assessment of the Level of Hydration in Studied Groups of Women

An important aspect of skin’s functioning is maintaining hydration, which is a variable factor that depends on age and lifestyle. It affects skin tension and provides elasticity [1,2,3,4].

Studies have shown that the use of mandelic acid, lactobionic acid, and cream resulted in different levels of skin hydration among women aged 30–40 years. The most effective hydrating preparation was lactobionic acid, the use of which resulted in an increased level of the examined parameter by 14.11 CK. In the group of older women, the treatment with lactobionic acid was similarly the most effective peel. In contrast, mandelic acid showed no statistical significance for the biophysical parameter studied.

It should be emphasized that in the study conducted in the control group, only a moisturizer was used, which included shea butter, d-panthenol, and silicones. These are very common ingredients in preparations recommended for daily facial skin care at home. It can be concluded that in a group of younger women, the systematic use of such a moisturizer produces similar results as a series of exfoliating treatments with mandelic and lactobionic acid. However, in older women, cosmetic treatments with lactobionic acid peel carried out in a beauty salon show better hydrating efficacy than the application of the moisturizer at home.

In a study carried out by Palacz A., the level of skin hydration after using 40% mandelic acid applied to women in three different age ranges was assessed. A series of appointments contained 6 treatments performed twice a week. The study and control group were made up of 30 people each. In women in the first age range (25–35 years), hydration improved by 12.90 CK; in the second age range (36–45), it improved slightly by 4.6 CK; and in the third age range (46–55 years) the hydration greatly improved by 12.23. In the study carried out, the control group consisted of women who used a moisturizer or nourishing cream, but the composition of these preparations was not specified. In participants aged 25–35 years, using moisturizer surprisingly caused their skin hydration to decrease by 1.65 CK; in the group aged 36–45 years, it decreased by 0.06, and in the third group (46–55 years), the greatest decrease was observed, which was equal to 2.40 CK. It is difficult to directly compare our own results with those of Palacz A., as the age ranges of participants and the number and frequency of treatments carried out do not match (in our study, treatments were carried out in accordance with the manufacturer’s recommendations). In the study of Palacz A., an increase in the level of skin hydration can be seen in all women taking part in the study after the use of 40% mandelic acid. However, in our study, skin hydration improved only in the group of younger women. Equally different results were obtained after the application of the moisturizer in the control group; namely, in the study of Palacz A., there was a decrease in the level of hydration among all participants studied, while in our study, this parameter increased in both age groups of women (30–40 years and 50–60 years).

In a study carried out by Sicińska A., the level of skin hydration in a group of 30 women aged 20–60 was assessed. In all of them, 6 exfoliating treatments using 50% mandelic acid were performed at 7-day intervals. After the exfoliating treatments, participants experienced an increase in hydration levels in two areas of facial skin. On the forehead, the parameter examined increased by 82%, while on the cheek, it increased by 77%. The results obtained cannot be directly related to our own results either, due to the number of women participating in the study, the lack of division into age groups, the number of exfoliating treatments, the different concentrations of mandelic acid, and the division of facial areas. However, it should be emphasized that in the study by Sicińska A., all women had a significant increase in the level of hydration in both areas of facial skin, whereas in our study, only the younger group of women had an increase in the level of the studied parameter [17].

In a study carried out by Algiert-Zielińska B. et al., the level of facial skin hydration was assessed in clients aged 26–73 years at a beauty salon after a series of 8 treatments with the “Split face” method using 10% and 30% lactobionic acid. The results showed that both concentrations of lactobionic acid increased the hydration level of the facial skin on which the procedures were performed. Another study by Algiert-Zielińska B. et al. assessed the effect of 20% lactobionic acid on the hydration parameter of facial skin in 20 clients who received 6 exfoliating treatments using the “Split face” method. The left cheek was treated with 20% lactobionic acid, and the epidermis was exfoliated with corundum microdermabrasion, while the right cheek was treated only with a peel containing 20% lactobionic acid. After the treatments, there was an increase in the level of hydration on both cheeks of the women’s facial skin, while better hydration was achieved on the left cheek, where, in addition to a chemical peel, a corundum microdermabrasion treatment was performed. Although in both studies by Algiert-Zielińska B. et al., the methodology of the treatments was different compared to our own study, in all cases, there was an increase in the level of skin hydration. It can be concluded that despite the different ages of the clients, the level of skin hydration improves after a series of treatments with lactobionic acid [18,19].

In the literature, studies assessing the effect of lactobionic acid used as a cream to hydrate the skin can be found. Such studies were carried out by Klauzińska O. et al., the purpose of which was to assess transepidermal water loss after regular use of a cream containing acid. The 30-person group of women aged 22–35 in whom inhibition of water loss from the epidermis and, thus, improvement in skin hydration was observed with regular home care. In our study, lactobionic acid was used in the form of a chemical peel applied at 7-day intervals. After a series of treatments with the acid, there was an increase in hydration levels in both younger and older women. It can be concluded that lactobionic acid used in various forms (peels, creams) improves skin hydration [20].

Our own and other authors’ results show that lactobionic acid hydrates the facial skin of younger and older people, which is in line with the claims of the manufacturers of these cosmetics. In contrast, this does not fully apply to mandelic acid, which improves hydration mainly in younger women. In the case of cosmetics with mandelic acid, the recipients should be mainly younger women in whom an improvement in hydration has been observed after a series of exfoliating treatments, or the series of treatments should be longer for older women.

### 5.2. Assessment of the Level of Flexibility in Studied Groups of Women

Another assessed biophysical parameter was facial skin elasticity in women aged 30–40 and 50–60 years. The skin, in the process of aging, becomes thinner and is less tense. The elasticity of the skin significantly deteriorates with age, but also with a decrease in body fat and increased muscle activity. Despite the progressive loss of elasticity with age, increased deformability, and brittleness, the skin retains its thickness and stretchability until around the age of 70. Increased expression of matrix metalloproteinases (MMPs), collagenase (MMP-1), gelatinase (MMP-2 and MMP-9), and elastase is responsible for biochemical changes in aging skin, including atrophy of collagen and elastin fibers. Collagen fibers are broken down by collagenases and gelatinases, while elastin fibers are broken down by elastases. It has also been found that the levels of MMP-1, which most often contributes to damage within collagen fibers, increase with age, which is considered one of the main factors in skin aging and loss of skin integrity [21,22,23,24,25,26].

In menopausal women, as a result of hormonal disorders and lack of stimulation of estrogen receptors within the facial skin, there is a decrease in the concentration of glycosaminoglycans, especially hyaluronic acid, which is responsible for optimal hydration. The consequence of hormonal disorders is the inhibition of the production of collagen, and hyaluronic acid and also a decrease in the synthesis, differentiation, and regeneration of keratinocytes [27].

In the study, a statistically significant improvement was observed in the elasticizing effect of the facial skin of women undergoing exfoliating treatments and in the control group. After the application of both acids and the moisturizer, there was an increase in the level of the studied parameter in younger and older women. The most effective method to improve facial skin elasticity among the studied younger women was the treatment using mandelic acid, while in the group of older women, it was lactobionic acid. It can be concluded that the systematic use of moisturizer also improves facial skin elasticity in both younger and older women but does not affect the elasticity parameter as significantly as a series of treatments with mandelic and lactobionic acid.

Palacz A. performed an analysis of the effect of 40% mandelic acid on the skin elasticity of 30 women aged 25–55 years, divided into three groups. The mandelic acid peel was applied twice a week, and the treatment series consisted of 6 appointments. The best results were obtained in women aged 36–45 years. In our study, the group of women aged 30–40 years also saw the greatest increase in this parameter. It should be noted that despite the different methodologies, in both cases, a beneficial effect of 40% mandelic acid on the parameter of facial skin elasticity of women undergoing exfoliating treatments was observed [16].

Algiert-Zielińska B. et al. studied the effect of 20% lactobionic acid on the parameter of facial skin elasticity of 20 beauty salon clients aged 26–73. Six exfoliating treatments were performed using the “Split face” method. A 20% lactobionic acid was applied to the left cheek, and the epidermis was exfoliated with corundum microdermabrasion, while the right cheek was treated with a peel with 20% lactobionic acid only. After the series of treatments, an increase in the level of elasticity was observed in both cheeks of the women’s facial skin. It should be noted that in the study by Algiert-Zielińska B. et al., as well as in our own study in both age ranges, the desired increase in elasticity occurred after using a lactobionic acid peel [19].

Studies by other authors have shown that lactobionic acid has an antioxidant effect and inhibits the activity of MMP-1, which leads to the preservation of collagen in the dermis and the maintenance of skin firmness and, thus, has an anti-aging effect [28,29].

Lactobionic acid has antioxidant properties that are associated with chelating iron and inhibiting the oxidation of other substances, which means that it helps reduce symptoms of skin aging associated with UV radiation [30].

In the literature, studies have assessed the effect on the elasticity of facial skin of mandelic and lactobionic acid used as a cream. The study by Jacobs S.W. et al. assessed the efficacy of using a cream containing mandelic acid for 4 weeks in terms of men’s and women’s (aged 42–68) facial skin elasticity. After application of the cream, there was a 23.8% increase in facial skin elasticity, while in the area of the lower eyelids alone, there was a 25.4% increase. In our study, the level of elasticity also increased in both groups of women; however, it should be noted that mandelic acid was applied in a series of 5 treatments at 7-day intervals. The studies cannot be directly compared with each other because they differ in the methodology used and the characteristics of the study groups [31].

Another study involving the cream was carried out by Green B.A. et al. The researchers assessed the effect of cream containing 8% lactobionic acid on the level of elasticity of women’s facial skin and epidermal thickening. The women in the study were 39–60 years old. The results showed that after 12 weeks of using the cream, there was an improvement in skin elasticity and thickening. According to the clients, lactobionic acid in the form of the cream improved the appearance of the skin, had an effect on its elasticity, and eliminated fine wrinkles around the eyes. In addition, histopathological examination showed an improvement in the cohesiveness of the stratum corneum, an increase in glycosaminoglycans, and a decrease in the amount of MMP-9, which is responsible for the breakdown of collagen and acceleration of skin aging [32].

In our own and other authors’ studies, both acids increased the level of skin elasticity regardless of the age of the participants. It can be concluded that mandelic and lactobionic acid, regardless of whether they are used in the form of cream or exfoliating treatments, are beneficial for the level of facial skin elasticity.

### 5.3. Limitations

The study involves only women aged 30–40 and 50–60, which limits the generalization of results to other age groups and men. The measurement of skin biophysical parameters was carried out only 14 days after the end of the series of treatments, which does not allow for the assessment of the long-term effects of the use of acids. Although the division of participants was random, factors such as diet, body hydration, or stress level, which may affect the condition of the skin, were not taken into account. All participants used the same moisturizing cream, but their previous care routine was not taken into account, which may have influenced the study results.

## 6. Conclusions

Based on the conducted researches, the following conclusions can be drawn.

1. The effectiveness of the impact of peels on the biophysical parameters of facial skin depends largely on the age of the surveyed women.
2. In the group of younger women, lactobionic acid significantly improved the level of facial skin hydration.
3. In the group of younger women, mandelic acid significantly improved the level of facial skin elasticity.
4. In the group of older women, mandelic acid significantly improved the level of elasticity.
5. In the group of older women, lactobionic acid significantly improved the level of elasticity and hydration.
6. Lactobionic acid should be dedicated to older women, while mandelic acid can be used interchangeably with lactobionic acid by younger women.

## Figures and Tables

**Table 1:** Hydration depending on the type of impact age 30–40 years.

Exhibition 30–40 Years	Type of Impact	*n*	M	SD	Me	Min	Max	Q1	Q3	*p*-Value
hydration before	mandelic acid	30	60.84	8.40	60.35	47.50	76.70	55.50	66.75	*p* = 0.001 *
hydration after	mandelic acid	30	67.49	8.49	69.58	50.15	91.50	61.65	73.00	
hydration before	lactobionic acid	30	56.64	11.02	57.25	33.00	77.50	50.35	64.85	*p* = 0.001 **
hydration after	lactobionic acid	30	70.75	11.15	69.58	49.65	95.00	64.45	76.80	
hydration before	control group	30	57.53	11.56	54.90	29.70	80.20	47.65	68.65	*p* = 0.001 **
hydration after	control group	30	67.05	9.22	68.33	50.05	87.15	58.50	73.05	

* Non-parametric test for two dependent samples *p* < α; α = 0.05; ** Student’s parametric *t*-test for dependent samples *p* < α; α = 0.05. Statistically significant.

**Table 2:** Elasticity depending on the type of impact age 30–40 years.

Exhibition 30–40 Years	Type of Impact	*n*	M	SD	Me	Min	Max	Q1	Q3	*p*-Value
elasticity before	mandelic acid	30	64.14	5.86	64.90	54.65	75.15	59.15	68.00	*p* = 0.000 *
elasticity after	mandelic acid	30	72.76	10.48	70.75	57.80	90.50	66.50	80.65	
elasticity before	lactobionic acid	30	62.94	7.27	61.75	46.20	88.50	58.85	66.35	*p* = 0.005 *
elasticity after	lactobionic acid	30	68.78	11.48	68.58	51.35	94.15	58.70	76.50	
elasticity before	control group	30	64.13	8.24	64.68	45.35	80.15	61.00	69.85	*p* = 0.046 **
elasticity after	control group	30	67.59	9.71	67.60	46.00	85.00	60.65	72.15	

* Wilcoxon paired *t*-test for dependent samples *p* < α; α = 0.05; ** Student’s parametric *t*-test for dependent samples *p* < α; α = 0.05.

**Table 3:** Facial skin hydration depending on the type of impact age 50–60 years.

Exhibition 50–60 Years	Type of Impact	*n*	M	SD	Me	Min	Max	Q1	Q3	*p*-Value
hydration before	mandelic acid	30	63.83	8.33	63.50	50.50	88.80	58.00	65.80	*p* = 0.497 *
hydration after	mandelic acid	30	62.84	8.21	64.25	48.50	76.35	58.00	70.00	
hydration before	lactobionic acid	30	59.95	10.77	61.83	39.00	79.90	52.05	68.35	*p* = 0.001 **
hydration after	lactobionic acid	30	71.27	9.98	70.45	52.50	90.50	62.65	79.50	
hydration before	control group	30	64.34	6.43	63.00	54.65	80.00	61.00	68.00	*p* = 0.014 **
hydration after	control group	30	67.68	10.95	65.75	48.40	85.50	63.00	78.80	

* Non-parametric test for two dependent samples *p* < α; α = 0.05; ** Student’s parametric *t*-test for dependent samples *p* < α; α = 0.05.

**Table 4:** Elasticity depending on the type of impact age 50–60 years.

Exhibition 50–60 Years	Type of Impact	*n*	M	SD	Me	Min	Max	Q1	Q3	*p*-Value
elasticity before	mandelic acid	30	62.00	6.41	64.00	51.00	70.50	55.20	67.00	*p* = 0.000 *
elasticity after	mandelic acid	30	68.54	9.16	69.03	52.00	90.50	60.20	72.50	
elasticity before	lactobionic acid	30	57.00	4.44	56.85	49.00	69.65	54.50	59.50	*p* = 0.000 **
elasticity after	lactobionic acid	30	64.40	6.82	63.93	46.85	77.00	61.00	68.85	
elasticity before	control group	30	58.68	6.05	59.55	49.50	68.50	53.00	64.65	*p* = 0.011 **
elasticity after	control group	30	62.67	7.70	62.20	48.00	74.00	56.70	69.00	

* Wilcoxon paired *t*-test for dependent samples *p* < α; α = 0.05; ** Student’s parametric *t*-test for dependent samples *p* < α; α = 0.05.

## Data Availability

The authors acknowledge that data supporting the conclusions of this study are available in this article. Derived data supporting the conclusions of this study are available from the corresponding author upon request.
